# LPS-Activated Monocytes Are Unresponsive to T4 Phage and T4-Generated *Escherichia coli* Lysate

**DOI:** 10.3389/fmicb.2016.01356

**Published:** 2016-08-31

**Authors:** Katarzyna Bocian, Jan Borysowski, Michał Zarzycki, Piotr Wierzbicki, Danuta Kłosowska, Beata Weber-Dąbrowska, Grażyna Korczak-Kowalska, Andrzej Górski

**Affiliations:** ^1^Department of Immunology, Faculty of Biology, University of WarsawWarsaw, Poland; ^2^Department of Clinical Immunology, Transplantation Institute, Medical University of WarsawWarsaw, Poland; ^3^Laboratory of Bacteriophages, Ludwik Hirszfeld Institute of Immunology and Experimental Therapy, Polish Academy of SciencesWrocław, Poland

**Keywords:** bacteriophage, T4, phage therapy, bacterial lysate, *Escherichia coli*, monocyte

## Abstract

A growing body of data shows that bacteriophages can interact with different kinds of immune cells. The objective of this study was to investigate whether T4 bacteriophage and T4-generated *Escherichia coli* lysate affect functions of monocytes, the key population of immune cells involved in antibacterial immunity. To that end, we evaluated how T4 and *E. coli* lysate influence the expression of main costimulatory molecules including CD40, CD80 and CD86, TLR2, TLR4 on monocytes, as well as the production of IL-6 and IL-12 in cultures of peripheral blood mononuclear cells (PBMCs). Separate experiments were performed on unactivated and LPS-activated PBMCs cultures. Both studied preparations significantly increased the percentage of CD14^+^CD16^-^CD40^+^ and CD14^+^CD16^-^CD80^+^ monocytes in unactivated PBMCs cultures, as well as the concentration of IL-6 and IL-12 in culture supernates. However, neither purified T4 nor *E. coli* lysate had any significant effect on monocytes in LPS-activated PBMCs cultures. We conclude that LPS-activated monocytes are unresponsive to phages and products of phage-induced lysis of bacteria. This study is highly relevant to phage therapy because it suggests that in patients with infections caused by Gram-negative bacteria the administration of phage preparations to patients and lysis of bacteria by phages are not likely to overly stimulate monocytes.

## Introduction

Bacteriophages are increasingly considered as a means of treatment of bacterial infections, including those caused by antibiotic-resistant bacteria (Międzybrodzki et al., 2012; Kutter et al., 2015). However, during treatment, phages can interact not only with bacteria, but also with different populations of immune cells including those involved in the induction of antibacterial immune responses (Górski et al., 2012). An important kind of immune cells engaged in antibacterial immunity are monocytes (Lauvau et al., 2015). These are cells of myeloid origin that constitute 5–12% of leukocytes in the peripheral blood in humans. The life span of monocytes in blood is up to 2 days. While unactivated monocytes undergo apoptosis, activation by different factors associated with ongoing infection or inflammation results in apoptosis inhibition, and monocytes migrate into inflamed tissues, phagocytose apoptotic cells, cellular debris, and different particles. In addition, monocytes can differentiate into macrophages and inflammatory dendritic cells (iDCs; Swirski et al., 2014). Along with DCs and macrophages, monocytes comprise a heterogeneous population of mononuclear phagocytes (MPS) involved in antigen processing and presentation to initiate and regulate immune responses to pathogens, vaccines, tumors, and tolerance to autoantigens. Main functions of the MPS system include tissue maintenance and healing, innate immunity and pathogen clearance, and the induction of adaptive immune responses (Reynolds and Haniffa, 2015).

Monocytes are one of the key populations of immune cells to combat bacterial infections. Apart from direct antibacterial effects mediated by phagocytosis, reactive oxygen species, and nitric oxide, monocytes are also involved in regulation of both innate and adaptive immune responses during infections (Lauvau et al., 2015). Given a central role of monocytes during bacterial infections, we investigated whether bacteriophages could affect functions of this population of cells. Because during ongoing infection monocytes can be activated by different components of bacterial cells including lipopolysaccharide (LPS; Salomao et al., 2012), we also included to our study experiments to investigate whether bacteriophages affect functions of LPS-activated monocytes. Our study focused on CD14^+^CD16^-^ classic monocytes, the most abundant subpopulation of these cells (Ziegler-Heitbrock, 2015).

The study was performed on T4 phage that for decades has been the model phage in various studies and has been extensively characterized at the genetic and molecular level (Leiman et al., 2003). Moreover, T4 was used in previous studies of the effects of phages on the immune system (Górski et al., 2012), as well as in the first randomized placebo-controlled safety test of phage therapy (Bruttin and Brüssow, 2005).

## Materials and Methods

### Bacteriophage

T4 phage was obtained from American Type Culture Collection (ATCC; USA) and was propagated on *E. coli* B from the Collection of Microorganisms of the L. Hirszfeld Institute of Immunology and Experimental Therapy (IIET), Wrocław, Poland.

Purified preparation of T4 phage was prepared by Laboratory of Bacteriophages, IIET, according to the protocol reported in detail by Boratyński et al. (2004). In brief, phage purification involved sequential ultrafiltration of crude T4 phage-generated *E. coli* B lysate through polysulfone membranes followed by chromatography on sepharose 4B (Sigma–Aldrich, Poland) and cellulofine sulfate (Millipore, USA). Stock preparations of T4 were suspended in phosphate-buffered saline (PBS; Biomed, Poland). Phage titer was measured by two-layer method of Adams (Adams, 1959).

The concentration of LPS in purified T4 phage preparation was determined using QLC-1000 Endpoint Chromogenic LAL test kit (Lonza, Switzerland) according to the manufacturer’s instructions. The concentration of LPS in the preparation was 3 ng/ml. Therefore, in immunological experiments, LPS (Sigma–Aldrich, Poland) diluted with PBS was used at a concentration of 3 ng/ml as an additional control for purified T4 phage preparation.

### Bacterial Lysate

T4 phage-generated bacterial lysate was prepared by Laboratory of Bacteriophages, IIET, according to the protocol reported by Slopek et al. (1983) and Letkiewicz et al. (2009). In brief, T4 was incubated with *E. coli* B in LB medium (Sigma–Aldrich, USA) at 37°C until complete bacterial lysis (approx. 4–6 h). Next the suspension was filtered through a 0.22-μm filter (Millipore, USA). Stock preparation of the lysate was suspended in peptone water (IIET, Poland). Phage titer in lysate was measured by two-layer method of Adams (Adams, 1959).

In immunological experiments an additional control for T4-generated *E. coli* lysate was peptone water.

### Cell Cultures

All experiments were performed on cells isolated from healthy blood donors. Informed, written consent was obtained from all donors. The study protocol was approved by the ethics committee of the Medical University of Warsaw. Peripheral blood mononuclear cells (PBMCs) were isolated from blood specimens by density-gradient centrifugation over Gradisol L (Aqua Medica, Poland). PBMCs were cultured at a density of 1 × 10^6^/ml in RPMI medium (Biomed, Poland) supplemented with FCS (Sigma–Aldrich, USA), L-glutamine (Sigma–Aldrich, USA), HEPES (Sigma–Aldrich, USA), and gentamicin (Krka, Slovenia) in 24-well plates at 37°C with 5% CO_2_ for 24 h. In each experiment, two parallel cultures were set up. In one culture, PBMCs were activated with LPS (Sigma–Aldrich; 10 μg/ml), and in the other cells were treated with equal volume of PBS. Simultaneously, in some cultures purified T4 phage (10^8^ PFU/ml; final concentration), *E. coli* lysate (containing T4 phage at the final concentration of 10^8^ PFU/ml), control LPS (3 ng/ml), or peptone water was also added to wells. In control cultures equal volume of PBS was added to wells. After 24 h of culture viability of PBMCs was determined using trypan blue. The viability of cells was consistently 95%. PBMCs were harvested for flow cytometry analysis of surface markers, and culture supernates were frozen at -20°C for measurements of cytokines concentrations.

### Evaluation of Expression of Monocytes Surface Markers

Cells were incubated with the following monoclonal antibodies: CD14-PerCP (BD Pharmingen, USA), CD16-FITC (BD Pharmingen), CD80-FITC (BD Pharmingen), CD86-PE (BD Pharmingen), CD40-PE (BD Pharmingen), TLR2-FITC (eBioscience, USA), and TLR4-PE (eBioscience). In isotype controls cells were stained with IgG1 conjugated with the respective fluorochromes (BD Pharmingen, eBioscience). After 30 min of incubation at 4°C, cells were washed twice in FACS buffer (Becton Dickinson, USA). The expression of monocytes surface markers was measured by flow cytometry (FACSCalibur, Becton Dickinson) and analyzed by Cell Quest software (Becton Dickinson). The results were based on analysis of at least 100,000 cells and were shown as the percentage of positively labeled cells. Moreover, the mean fluorescence intensity (MFI) values of gated monocytes positive for individual markers were determined.

### Determination of Cytokine Production

The concentrations of IL-6 and IL-12 (p70) were measured in cell culture supernates by enzyme-linked immunoassay (ELISA) using Human IL-6 and IL-12 p70 ELISA Ready-SET-Go kits (eBioscience) according to the manufacturer’s instructions.

### Statistics

Statistical analysis of the results was performed by Wilcoxon’s matched pairs test. *P* < 0.05 was considered significant.

## Results

In order to investigate the effects of T4 bacteriophage and T4-generated *E. coli* lysate on monocytes, we determined: (1) the expression of main costimulatory molecules including CD40, CD80, and CD86, (2) the expression of TLR2 and TLR4 molecules, and (3) the production of IL-6 and IL-12. In each case, separate experiments were performed on unactivated and LPS-activated PBMCs.

**FIGURE 1:**
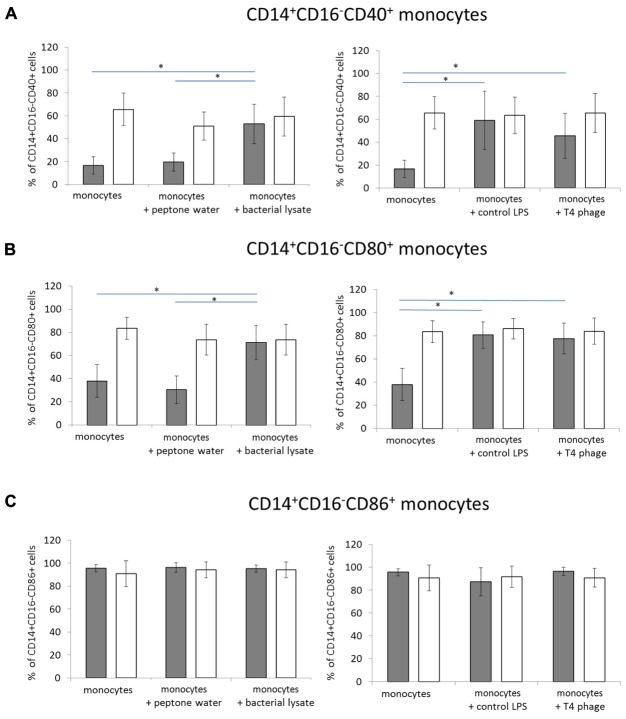
**The effects of T4 phage and T4-generated *Escherichia coli* lysate on the expression of costimulatory molecules on monocytes.** The percentage values of CD40^+^ **(A)**, CD80^+^ **(B)**, and CD86^+^ **(C)** cells were determined by flow cytometry in CD14^+^CD16^-^ monocytes from 24-h cultures of peripheral blood mononuclear cells (PBMCs). The expression of each marker was determined separately on monocytes from cultures of unactivated (gray bars) and LPS-activated (white bars) PBMCs. T4 phage or *E. coli* lysate was present in culture medium throughout the culture. Shown are the mean percentage values ± standard deviation. Statistically significant differences (*p* < 0.05) are marked with ^∗^.

In the first set of experiments, we evaluated whether T4 bacteriophage and T4-generated *E. coli* lysate affect the expression of CD40, CD80, and CD86 in CD14^+^CD16^-^ monocytes. In unactivated PBMCs cultured with T4 phage, the mean percentage of CD14^+^CD16^-^CD40^+^ monocytes was 45.47 ± 19.5, while in the control (unactivated PBMCs to which equal volume of PBS was added) this percentage was 16.55 ± 7.61 (*p* = 0.016; **Figure 1A**). However, a comparable increase in the mean percentage of CD14^+^CD16^-^CD40^+^ cells was observed in unactivated PBMCs cultured with control LPS (58.99 ± 25.39); the difference between the mean percentage of CD14^+^CD16^-^CD40^+^ monocytes in cultures to which T4 phage or control LPS was added was not statistically significant. Similarly to T4 phage, T4-generated *E. coli* lysate significantly increased the mean percentage of CD14^+^CD16^-^CD40^+^ monocytes to 52.81 ± 17.02 (*p* = 0.008 compared with the control). On the other hand, we found no significant differences between the MFI values of CD40 in CD14^+^CD16^-^ monocytes from PBMCs cultures to which PBS, T4 phage, control LPS, or *E. coli* lysate was added (**Table 1**). In LPS-activated PBMCs cultures, the percentage of CD14^+^CD16^-^CD40^+^ monocytes was comparable in all culture variants (65.6 ± 14.17, 65.49 ± 17.1, 63.49 ± 15.87, and 59.37 ± 37 for variants to which PBS, T4 phage, control LPS, or *E. coli* lysate was added, respectively). Furthermore, in LPS-activated PBMCs cultures the MFI values of CD40 in CD14^+^CD16^-^ monocytes were also comparable in all culture variants (**Table 1**).

**Table 1:** The mean fluorescence intensity (MFI) of costimulatory molecules and TLRs on monocytes cultured with T4 phage and T4-generated *Escherichia coli* lysate.

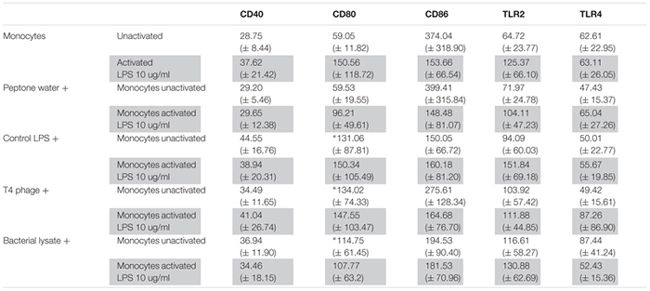

*The MFI of costimulatory molecules (CD40, CD80, CD86), TLR2, and TLR4 was determined by flow cytometry in CD14^+^CD16^-^ monocytes from 24-h cultures of peripheral blood mononuclear cells (PBMCs). All experiments were performed on unactivated PBMCs (results in white boxes) and LPS-activated PBMCs (results in gray boxes). T4 phage or *E. coli* lysate was present in culture medium throughout the culture. Shown are the MFI values ± standard deviation. Statistically significant differences (*p* < 0.05) are marked with ^∗^.*

Moreover, we found that T4 phage significantly increased the mean percentage of CD14^+^CD16^-^CD80^+^ monocytes in unactivated PBMCs cultures compared with the control (77.55 ± 13.31 and 37.91 ± 14.06 for cells cultured in the presence of T4 and control cells, respectively; *p* = 0.0006; **Figure 1B**); however, a similar increase in the mean percentage of CD14^+^CD16^-^CD80^+^ monocytes was observed in cultures to which control LPS was added (80.61 ± 11.38). Like T4 phage, *E. coli* lysate significantly increased the mean percentage of CD14^+^CD16^-^CD80^+^ monocytes (71.2 ± 14.75; *p* = 0.0019 compared with the control). In addition, we found that in unactivated PBMCs cultures, T4, control LPS, and *E. coli* lysate significantly increased the MFI values of CD80 in CD14^+^CD16^-^ monocytes compared with the control (*p* = 0.0063, *p* = 0.00238, and *p* = 0.0379 for T4 phage, control LPS, and *E. coli* lysate, respectively; **Table 1**).

In LPS-activated PBMCs, the mean percentage of CD14^+^CD16^-^CD80^+^ monocytes was comparable in cultures to which PBS, T4 phage, control LPS, or *E. coli* lysate was added (95.61 ± 3.16, 96.41 ± 3.55, 87.26 ± 12.38, and 95.17 ± 3.13, respectively; **Figure 1B**). Furthermore, in LPS-activated PBMCs, we found no statistically significant differences between the MFI values of CD80 in CD14^+^CD16^-^ monocytes from individual culture variants (**Table 1**).

We also found that neither T4 phage nor *E. coli* lysate had any significant effect on the mean percentage value of CD14^+^CD16^-^CD86^+^ monocytes in unactivated PBMCs; these values were 90.8 ± 11.3, 83.3 ± 11.31, and 73.56 ± 13.25 in cultures to which PBS, T4 phage, or lysate was added (**Figure 1C**). The corresponding MFI values were also comparable in all culture variants (**Table 1**). Likewise, in LPS-activated PBMCs, we found no significant differences between the mean percentage of CD14^+^CD16^-^CD86^+^ monocytes in individual culture variants (73.56 ± 13.25, 90.87 ± 8.0, and 94.33 ± 6.87 in cultures to which PBS, T4 phage, or *E. coli* lysate was added, respectively; **Figure 1C**). In addition, we found that in LPS-activated PBMCs neither of the studied preparations significantly affected the MFI value of CD86 in CD14^+^CD16^-^ monocytes (**Table 1**).

**FIGURE 2:**
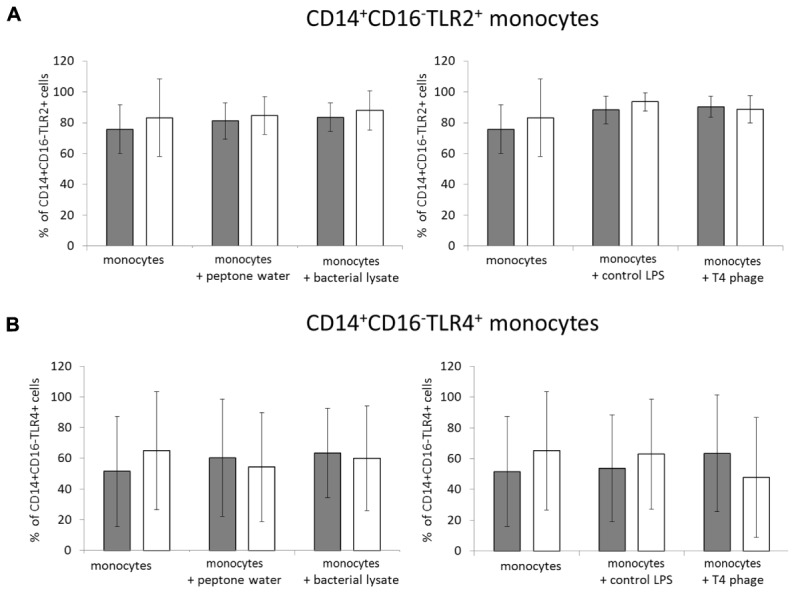
**T4 phage and T4-generated *Escherichia coli* lysate do not affect the expression TLR2 and TLR4 on CD14^+^CD16^-^ monocytes.** The percentage values of TLR2^+^ **(A)** and TLR4^+^ **(B)** cells were determined by flow cytometry in CD14^+^CD16^-^ monocytes from 24-h cultures of PBMCs. The expression of both markers was determined separately on monocytes from cultures of unactivated (gray bars) and LPS-activated (white bars) PBMCs. T4 phage or *E. coli* lysate was present in culture medium throughout the culture. Shown are the mean percentage values ± standard deviation.

In the second set of experiments, we evaluated whether T4 phage and *E. coli* lysate affect the expression of TLR2 and TLR4 on CD14^+^CD16^-^ monocytes. Overall, neither of the studied preparations significantly affected the expression of TLR2 or TLR4. In unactivated PBMCs the mean percentage of CD14^+^CD16^-^TLR2^+^ cells was comparable in all culture variants (75.81 ± 15.87, 90.42 ± 6.8, and 83.54 ± 9.21 in cultures to which PBS, T4 phage, or lysate was added; **Figure 2**). In LPS-activated PBMCs the corresponding values were 83.21 ± 25.21, 88.69 ± 8.8, and 87.98 ± 12.71. The mean percentage of CD14^+^CD16^-^TLR4^+^ cells in unactivated PBMCs cultures was 51.53 ± 35.74, 47.73 ± 39.01, and 60.02 ± 34.02 in control cultures and cultures to which T4 phage or lysate was added (**Figure 2**). In LPS-activated PBMCs these values were 65.04 ± 38.4, 47.73 ± 39.01, and 60.02 ± 34.02.

**FIGURE 3:**
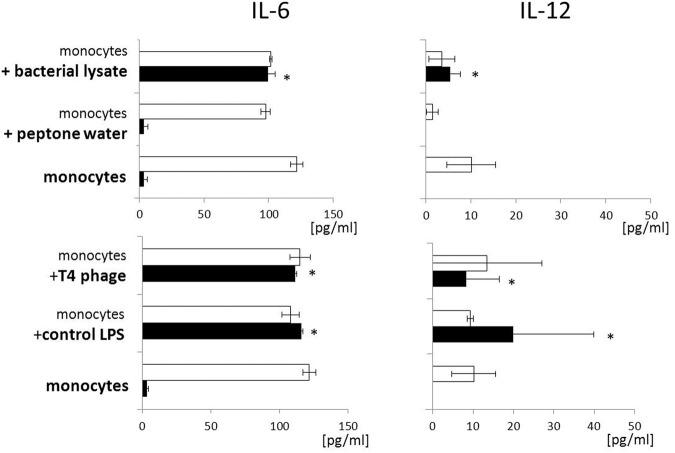
**T4 phage and T4-generated *Escherichia coli* lysate have no effect on the production of pro-inflammatory cytokines by LPS-activated PBMCs.** Concentration of pro-inflammatory cytokines – IL-6 **(Left)** and IL-12 **(Right)** – was measured by ELISA in supernates from 24-h cultures of PBMCs. Production of both cytokines was evaluated separately in cultures of unactivated (black bars) and LPS-activated (white bars) PBMCs. T4 phage or *E. coli* lysate was present in culture medium throughout the culture. Shown are the mean concentrations ± standard deviation. Statistically significant differences (*p* < 0.05) are marked with ^∗^.

Moreover, we evaluated whether the investigated preparations stimulate the production of pro-inflammatory cytokines IL-6 and IL-12. We found that both T4 phage and *E. coli* lysate significantly increased the concentration of IL-6 in supernates of unactivated PBMCs cultures compared with the control (the mean concentration of IL-6 3.47 ± 2.62, 111.58 ± 15.11, and 99.68 ± 5,32 pg/ml in control cultures and cultures to which T4 or lysate was added, respectively; *p* = 0.0294 for both T4 and lysate; **Figure 3**). A comparable increase in IL-6 concentration was observed in supernates of unactivated PBMCs cultures to which control LPS was added (115.99 ± 10.86 pg/ml; *p* = 0.0294 compared with the control; **Figure 3**). In LPS-activated PBMCs cultures the mean IL-6 concentration exceeded 100 pg/ml and was comparable in all culture variants (**Figure 3**).

Similar results were obtained for IL-12. Both T4 and *E. coli* lysate significantly increased the concentration of this cytokine in supernates of unactivated PBMCs cultures compared with the control (mean IL-12 concentrations 0.0, 8.22 ± 4.25, 5.42 ± 2.25 pg/ml in control cultures and cultures to which T4 or lysate was added, respectively; *p* = 0.211 for both T4 and lysate; **Figure 3**). IL-12 concentration was also significantly increased in supernates of unactivated PBMCs cultured with control LPS (19.92 ± 8.75 pg/ml; *p* = 0.0211 compared with the control; **Figure 3**). In supernates of LPS-activated PBMCs cultures the concentration of IL-12 was above 10 pg/ml and, we found no significant differences between individual culture variants (**Figure 3**).

## Discussion

The objective of this study was to evaluate whether bacteriophages can affect functions of monocytes, a key population of immune cells involved in antibacterial immunity (Lauvau et al., 2015). We focused on CD14^+^CD16^-^ classic monocytes, the most abundant subpopulation of monocytes (Ziegler-Heitbrock, 2015). All experiments were performed on T4 phage, a model dsDNA bacterial virus that has been extensively characterized at the genetic and molecular level (Leiman et al., 2003). Apart from purified preparation of T4, we used T4-generated *E. coli* lysate. *In vitro* experiments involving use of lysate were intended to mimic the effects of lysis of bacteria in a patient treated with a phage preparation. Thus, by using lysate, we investigated whether monocytes functions can be affected by products of phage-induced lysis of bacterial cells. Moreover, lysates are used in the treatment of bacterial infections at some centers of phage therapy (Międzybrodzki et al., 2012).

All experiments were performed on both unactivated and LPS-activated PBMCs. LPS is the principal component of Gram-negative bacterial cells known to activate monocytes in different infections including sepsis (Salomao et al., 2012). Experimental variants involving LPS-activated monocytes were included to evaluate whether bacteriophages can affect functions of monocytes that are in a state corresponding to that found in patients with ongoing bacterial infection. Recent microarray-based gene expression studies confirmed that in patients with bacterial infections, especially sepsis, PBMCs are activated, as reflected by up-regulation of many genes involved in antibacterial immunity and inflammatory responses (Maslove and Wong, 2014; Severino et al., 2014).

We found that both purified T4 and *E. coli* lysate significantly increased the percentage of CD14^+^CD16^-^CD40^+^ and CD14^+^CD16^-^CD80^+^ monocytes in unactivated PBMCs cultures without any significant effect on the percentage of CD14^+^CD16^-^CD86^+^ monocytes. However, we also observed a significant increase in the percentage of both CD14^+^CD16^-^CD40^+^ and CD14^+^CD16^-^CD80^+^ monocytes in unactivated PBMCs cultured with control LPS (used at a concentration of 3 ng/ml that corresponds to that found in T4 phage preparation). These findings suggest that the increase in the percentage of CD14^+^CD16^-^CD40^+^ and CD14^+^CD16^-^CD80^+^ monocytes observed in unactivated PBMCs cultured with purified T4 preparation was caused by residual LPS rather than bacteriophage virions themselves.

CD40, a member of the TNF receptor family, plays an important role in both cellular and humoral immune responses. Signaling through CD40 up-regulates the expression of a number of molecules including MHC class II, CD80, CD86, adhesion molecules and induces the production of cytokines such as IL-1, IL-6, IL-10, and TNF-α (Wu et al., 2009). Thus, our results suggest that in unactivated monocytes some products of phage-induced lysis of bacterial cells, especially LPS, may promote CD40-mediated humoral and cellular immune responses.

CD80 is a molecule whose expression on monocytes is increased following stimulation by some factors including LPS. CD80 can prolong or enhance costimulatory signals transmitted from antigen-presenting cells (APCs) to CD4^+^ T cells thus promoting the activation of these cells (Rutkowski et al., 2003). Therefore, our data indicate that LPS, and possibly some other components of *E. coli* cells released from bacteria during phage-induced lysis can promote CD80-mediated costimulation of CD4^+^ T cells.

Importantly, we also found that in LPS-activated PBMCs cultures the percentage of CD40, CD80, and CD86 on CD14^+^CD16^-^ monocytes was unaffected by purified T4 and *E. coli* lysate. These results suggest that in patients with infections caused by Gram-negative bacteria the administration of phage preparations, as well as lysis of bacteria by phage, is not likely to affect CD40-, CD80-, and CD86-mediated functions of monocytes.

We also showed that neither of the studied preparations significantly affected the expression of TLR2 and TLR4 on CD14^+^CD16^-^ monocytes both in unactivated and LPS-activated PBMCs cultures. TLRs are the key class of pattern-recognition receptors involved in the induction of innate immune responses to various pathogens (De Nardo, 2015). Thus our results suggest that neither phage nor products of lysis of bacterial cells are likely to affect TLR2- and TLR4-mediated immune responses.

Moreover, we showed that both T4 and *E. coli* lysate significantly increased the concentration of IL-6 and IL-12 in supernates of unactivated PBMCs cultures. While IL-6 and IL-12 are generally classified as “proinflammatory,” in fact their actual biological effects are highly context-dependent and both cytokines may even exert anti-inflammatory activity (Chang and Radbruch, 2007; Hunter and Jones, 2015). Our previous observations indicate that lysates used for therapeutic purposes do not induce any proinflammatory effects in patients. For example, lysates did not increase leukocytosis, sedimentation rate, and the concentration of C-reactive protein (CRP) in patients; furthermore, in individuals with the initial CRP concentration above 10 mg/l, a significant reduction in the concentration of this protein was shown between days 9 and 32 of treatment (Międzybrodzki et al., 2012). In line with our previous findings are the results of the present study that show that neither T4 nor *E. coli* lysate significantly increased the production of IL-6 and IL-12 in LPS-activated PBMCs cultures.

An important question is whether our results obtained for T4 phage and *E. coli* lysate can be extrapolated to other phages and bacterial lysates. At the present state of research it is hard to formulate general conclusions especially with respect to phages because it is known that structure of tailed phages is highly diversified and substantial differences in amino-acid sequences of phage proteins, including surface capsid proteins have been reported (Fokine and Rossmann, 2014). Therefore, it is possible that some phages have proteins that can activate some functions of monocytes. With respect to bacterial lysates, we believe that some differences between the effects of Gram-negative and Gram-positive bacteria might be expected due to different structure and composition of their cell walls (Silhavy et al., 2010). Thus, further studies should be conducted to evaluate whether activated monocytes are also refractory to other phages and phage-generated bacterial lysates.

## Conclusion

While LPS, and possibly some other products of phage-induced lysis of *E. coli* affect the phenotype of unactivated monocytes and production of cytokines by unactivated PBMCs, LPS-activated cells are unresponsive to both phage virions and products of phage-induced lysis of bacterial cells. Our results are of importance to phage therapy because they suggest that the administration of phage preparations to patients with infections caused by Gram-negative bacteria and subsequent lysis of bacteria by phages are not likely to overly stimulate monocytes.

## Author Contributions

KB performed the experiments, analyzed data, and contributed to drafting of the manuscript. JB analyzed data and contributed to drafting of the manuscript. MZ, PW, and DK performed the experiments. BW-D analyzed data. GK-K conceived the study, analyzed data and contributed to drafting of the manuscript. AG conceived the study and contributed to drafting of the manuscript. All authors approved the final version of the manuscript.

## Conflict of Interest Statement

AG and BW-D are co-inventors on patents covering preparation of therapeutic phages owned by the IIET, Wrocław, Poland. The institutions that funded the study had no role in the design of the study; in the collection, analyses, or interpretation of data; in the writing of the manuscript, and in the decision to publish the results. Neither the authors nor their institutions received payment or services from a third party for any aspect of the submitted work. All the other authors declare that the research was conducted in the absence of any commercial or financial relationships that could be construed as a potential conflict of interest.
